# A Comparative Study on the Routing Problem of Electric and Fuel Vehicles Considering Carbon Trading

**DOI:** 10.3390/ijerph16173120

**Published:** 2019-08-27

**Authors:** Wenzhu Liao, Lin Liu, Jiazhuo Fu

**Affiliations:** 1 School of Management Science and Real Estate, Chongqing University, Chongqing 400044, China; 2 College of Mechanical Engineering, Chongqing University, Chongqing 400044, China

**Keywords:** electric vehicle, routing problem, hybrid genetic algorithm, carbon prices, carbon quotas

## Abstract

In order to explore the impact of using electric vehicles on the cost and environment of logistics enterprises, this paper studies the optimization of vehicle routing problems with the consideration of carbon trading policies. Both the electric vehicle routing model and the traditional fuel vehicle routing model are constructed aiming at minimizing the total costs, which includes the fixed costs of vehicles, depreciation costs, penalty costs for violating customer time window, energy costs and carbon trading costs. Then a hybrid genetic algorithm (HGA) is proposed to address these two models, the advantages of greedy algorithm and random full permutation are combined to set the initial population, at the same time, the crossover operation is improved to retain the excellent gene fragments effectively and the hill climbing algorithm is embedded to enhance the local search ability of HGA. Furthermore, a case data is used with HGA to carry out computational experiments in these two models and the results indicate that first using electric vehicles for distribution can indeed reduce the carbon emissions, but results in a low customer satisfaction compared with using fuel vehicles. Besides, the battery capacity and charge rate have a great influence on total costs of using electric vehicles. Second, carbon price plays an important role in the transformation of logistics companies. As the carbon price changes, the total costs, carbon trading costs, and carbon emissions of using electric vehicles and fuel vehicles are affected accordingly, yet the trends are different. The changes of carbon quota have nothing to do with the distribution scheme and companies’ transformation but influence the total costs of using electric and fuel vehicles for distribution, and the trends are the same. These reasonable proposals can support the government on carbon trading policy, and also the logistics companies on dealing the relationship between economic and social benefits.

## 1. Introduction

In recent years, the environmental pollution and energy shortage have attracted the attention of all countries in the world. To cope with this challenge, most countries have developed relevant carbon policies, such as carbon cap and trade, carbon tax, and carbon quota. EU has implemented carbon cap and trade from 2005 and imposed carbon tax since 2012. Therefore, how to effectively reduce carbon emissions and save energy has become an urgent task to solve the world’s sustainable development. In 2005, the Chinese government made a promise to reduce carbon dioxide emissions per unit of GDP by 40–45% by 2020 at the Copenhagen Climate Summit [1]. With the rapid development of industry and economy, transportation has become the main source of carbon emissions. According to statistics, Chinese logistics industry’s carbon emissions account for nearly one-fifth of the total social emissions, far exceeding that of Europe and the United States. China bears enormous energy-saving, emission reduction pressures and social responsibilities. Thus, achieving low-carbon logistics is an important part of achieving a low-carbon economy [2].

Compared with fuel vehicles, electric vehicles have many advantages, such as almost zero emissions, high energy efficiency, and low noise [3]. China government has released relevant policies to encourage transportation enterprises to adopt electric vehicles to replace traditional fuel vehicles. On the one hand, with the development of renewable energy such as wind energy and solar energy, the use of electric vehicles for logistics distribution activities can effectively alleviate the dependence of traditional vehicles on non-renewable energy such as fossil fuels [4]. On the other hand, reasonable path planning can effectively reduce the distance traveled by vehicles and the emission of hazardous gases, improve the use efficiency of distribution vehicles, and reduce environmental pollution [5,6,7,8]. Many enterprises are on the way of replacing fuel vehicles with electric vehicles, such as JD said in 2018 that all delivery trucks would be replaced with electric vehicles in 2 years, Green hand released the new energy intelligent logistics vehicle plan in 2017 and the goal was to create 1 million electric vehicles for logistics express delivery activities in 5 years. Therefore, it is an inevitable trend for the transportation industry to adopt electric vehicles with high energy efficiency and low noise to replace traditional fuel vehicles [9].

In addition, although using electric vehicles is very environment-friendly, there are some non-negligible problems, such as short range, long charging time, and relatively high price [10], which resulted in the difficulties when enterprises are trying to adopt electric vehicles for transportation. Moreover, traditional vehicle routing models do not take these factors into consideration, and thus how to construct electric vehicle routing model to make reasonable path planning becomes extremely important, such as the determination of charging station and charging time.

Carbon trading is proved to be an effective and important tool for reducing carbon emissions [10,11,12]. Carbon dioxide emissions are used as commodity to form carbon dioxide emissions trading, known as carbon trading. Although electric vehicles do not produce any hazardous gases, carbon dioxide generated by a thermal power plant when generating electricity. In China, the main source of electricity is still based on thermal power, according to China’s land transport enterprises greenhouse gas emissions calculation methods and reporting guidelines released by the National Development and Reform Commission, the indirect carbon emissions include using electricity which also need to be considered. Thus, those enterprises using electric vehicles for transportation also have emitted a certain amount carbon dioxide.

In summary, this paper studies the Capacitated Electric Vehicle Routing Problem with Time Windows (CEVRPTW) and Capacitated Fuel Vehicle Routing Problem with Time Windows (CVRPTW), combining the present carbon trading policy and set the aim of minimum total cost to explore the impact of using electric vehicle for logistics enterprises under new energy situation, which could help those logistics enterprises to make a transformation and the government to make relevant carbon trading policy. Moreover, this paper would solve the following questions: How to quantify carbon emissions of electric vehicles; how to establish CEVRPTW and CVRPTW considering carbon trading; how the total costs and carbon emissions change when the relevant parameters of electric vehicles and carbon price and quota change.

## 2. Literature Review 

Since this paper is first to obtain a better solution for CEVRPTW and CVRPTW and then to make a comparison of total costs and carbon emission while carbon price and quotas change. The literature about vehicle routing problem (VRP) are reviewed in the following areas: electric vehicle routing problem (EVRP) and traditional VRP considering carbon emissions.

### 2.1. Electric Vehicle Eouting Problem

Despite the rapid development of electric vehicle in recent years, many researchers focused on electric vehicle technology itself, such as battery [13,14], body material [15], or charge station location problem [16,17]. Only a few scholars studied EVRP. Thus, the research about EVRP considering carbon trading is much less. Artmeirer et al. [18] firstly introduced electric vehicles to VRP in 2010, planning an economical path from the perspective of energy consumption. Schneider et al. [19] studied EVRP with time windows, assumed that the charging function of electric vehicle was linear and the vehicle must be fully charged while leaving the charging station. Bruglieri et al. [20] and Keskin et al. [21] allowed the vehicle to be partially charged at the charging station in their studies. In terms of charging methods, some scholars studied chargers of different specifications. For example, Keskin et al. [22] studied EVRP with time windows, considered the charging stations could be equipped with chargers of different specifications, and explored the influence of different charging methods on delivery time. Desaulniers et al. [23] used an exact algorithm to solve EVRP, but assumed that the battery charge level was a linear function of charging time. In 2017, Montoya et al. [24] firstly established an EVRP model considering nonlinear charging function and solved it with a mixed meta heuristic algorithm. The results showed that ignoring the nonlinear charging process of electric vehicles might result in a distribution scheme that was not feasible or costly. Forger et al. [25] established a nonlinear charging function with limited number of chargers, considering the nonlinear variation of battery charge level and charging time. Keskin et al. [26] studied EVRP with time windows considering the queuing time at recharging stations and proposed a metaheuristic combining adaptive large neighborhood search with an exact method. The results showed that queuing times might affect routing and charging decisions. However, all these researches assumed that the power consumption rate was constant (i.e., the power consumption rate per unit distance was the same), which ignoring the influence of vehicle load, traveling speed, and other relevant factors on electric energy consumption. Obviously, it does not meet the actual delivery process. Hence, this paper considers the actual power consumption of electric vehicles in the distribution process.

In addition, the objective functions of EVRP are mainly focused on costs. Shao et al. [27] studied EVRP with the aim of minimum total cost including vehicle fixed cost, total time cost, and charging cost. Hiermann et al. [28] proposed a hybrid genetic algorithm based on hierarchical path evaluation for solving EVRP with the aim to minimize vehicle fixed cost, charging cost, and power consumption cost. Paz et al. [29] studied the multiple-depot electric vehicle location routing problem, taking the vehicle fixed cost and travel cost into consideration. Macrina et al. [30] researched on the hybrid fleet path problem of electric vehicles and traditional fuel vehicles with the goal of minimum total travel cost and charging cost. Grandinetti et al. [31] studied EVRP with soft time windows and take penalty cost of violation of time windows into total cost. Froger et al. [32] set the aim of minimizing the total driving and charging time to solve EVRP with nonlinear charging function. Similarly, Koç et al. [33] introduced EVRP with shared charging stations considering nonlinear charging function to minimize total costs of opening charging stations and total driver costs.

From the perspective of selection about objective function of EVRP research, it could be seen that these studies aimed at achieving lowest cost or shortest time, with no attention to social benefit. Once a country’s energy structure mainly depends on thermal power generation, indirect carbon emission of using electricity cannot be neglected. Therefore, this paper considers indirect carbon emission and analyzes the impact of using electric vehicles on logistics companies under carbon trading market.

### 2.2. Vehicle Routing Problem (VRP) Considering Carbon Emission

With more concern for environment issues, many scholars took energy consumption and carbon emission into consideration in VRP models. The objective functions of VRP models considering carbon emission could be divided into two types: (1) convert carbon emission to costs for economical and social benefit and minimize the total costs; (2) minimize carbon emission just for social benefit.

In the first case, Kwon et al. [34] studied the heterogeneous fixed fleet VRP considering carbon trading and adopted a tabu search algorithm to minimize the total costs. The result showed that the amount of carbon emission could be reduced without increasing total costs by carbon trading. Wang et al. [1] studied VRP with time windows for cold-chain logistics and analyzed the influence of carbon tax on routing planning and carbon emission. Then, Wang et al. [35] studied the location-routing problem for cold chain logistics considering carbon tax. Also, Wang et al. [36] studied the inventory routing problem in refined oil logistics with the perspective of carbon tax. Shen et al. [37] studied multi-depot open VRP with time windows considering carbon trading. Niu et al. [38] investigated green open VRP with time windows to minimize the total costs, which included fuel costs, carbon emissions cost, and driver costs. Qin et al. [39] combined customer satisfaction and carbon emission and the result showed that there was a trade-off between them. Kim et al. [40] studied the impact of carbon trading on carbon emissions in multi-period heterogeneous VRP. Li et al. [7] studied the heterogeneous fixed fleet VRP by minimizing total costs, which included fixed expenses, fuel consumption, and carbon emissions costs.

In summary, although there are some researches about VRP considering relevant policies, such as carbon tax or carbon trading. There is little research that has paid attention to EVRP under carbon trading, particularly the comparative research of VRP and EVRP based on carbon trading. Thus, this paper firstly proposes a CEVRPTW model to optimize distribution paths and analyzes the impact of electric vehicles’ relevant parameters on total costs. Then, in order to make a comparison, a CVRPTW model is constructed and the impact of variation of carbon price or quota on total costs of two models is analyzed, which could greatly support the decisions making of logistics enterprises on adopting electric vehicles or not.

Due to poor local search ability of genetic algorithms, this paper develops an insertion algorithm based on time series to improve the quality of initial population, and then a random permutation method is used to enhance the population diversity. In addition, this paper adopts a tournament selection strategy for the selection operator design to maximize the individuality of good individuals, and also improves traditional crossover operator to ensure that the children of two good individuals can inherit the parents’ excellent sub-paths to a greater extent, as well as increasing the diversity of offspring. Finally, the paper embeds a hill-climbing operator in HGA, and performs the hill-climbing operation for best individual of each generation to improve algorithm convergence effectively.

## 3. Model Construction

This section firsts introduce the problem description of CEVRPTW and CVRPTW, then analyzes the corresponding sub-costs of these two models. Finally, the specific formulations of CEVRPTW and CVRPTW are presented.

### 3.1. Problem Description

**Figure 1:**
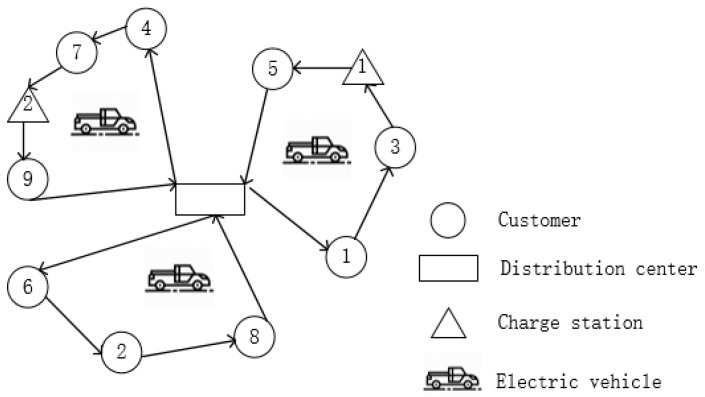
A simplified diagram of electric vehicle routing problem (EVRP).

For CEVRPTW, there is a distribution center with a certain number of electric vehicles, and a set of customers to be served. The locations of the center, charging stations, and customers are known, and the demands of each customer are also known. When the transportation tasks are completed, all the vehicles must return to the distribution center. In traditional VRP network, the node set is a collection of customer nodes and distribution center, and all customer nodes can only be visited once. However, in EVRP network, the node set also includes charging station nodes which could be visited twice or more. The schematic diagram is shown in Figure 1. The main purpose is to find an optimal solution considering factors of cost and carbon emission and make a comparison with fuel vehicles scheduling scheme under carbon trading market. The detailed assumptions are given as follows:

(1) The electric vehicles are homogeneous and the battery is fully charged when departing from the distribution center;
(2) Each electric vehicle has a limited load capacity and the total demand of the customers cannot exceed the total load of the fleet;
(3) The electric vehicles can only charge when arriving at the distribution center or charging stations;
(4) The electric vehicles will go to the charging stations only when they do not have enough energy to reach next customer;
(5) When recharging is undertaken, the batteries are filled to capacity and the charging time of electric vehicles is a fixed value.

For CVRPTW, the main differences about problem description compared with CEVRPTW is that the refueling process of fuel vehicles is not considered in the distribution process, hence the charging station nodes are not included in the note set.

The assumptions of CVRPTW are the same with CEVRPTW after relaxing the battery capacity constraint, such as, the vehicles need to be homogeneous and go back to the distribution center after the task is completed. In short, CEVRPTW is the same with CVRPTW if the battery capacity is a great positive value.

### 3.2. Notations

Based on the demands of model construction, the notations in CEVRPTW and CVRPTW of this paper are listed in Table 1. 

### 3.3. CEVRPTW Model

The CEVRPTW model in this paper is proposed to realize the optimal path selection from the perspective of both economic and social benefit. Carbon emissions generate from the thermal power plant to produce electricity, in other words, the lowest carbon emissions scheduling scheme is the least energy consumption. However, if only the minimum carbon emissions or energy consumption is set as the optimization goal, it is pointless for logistics enterprises. Moreover, with the rapid improvement of national carbon trading market, the land transportation companies are concerned about whether they need to use new energy vehicles under carbon trading market. Therefore, the objective function in this paper is not to minimize carbon emissions or electricity consumption, but to minimize total costs.

The total costs include fixed costs of electric vehicles, depreciation costs, electricity consumption costs, and penalty costs for violating customer time window and carbon emission trading costs.

(1) The fixed costs of electric vehicles

When dispatching the vehicles to carry out the distribution task, some fixed costs must be paid, including the drivers’ salary, the vehicle wear and tear and road maintenance fees. Thus, the fixed cost $C_{1}$ can be expressed as:

(1) $$C_{1}=\sum_{k\in K}f_{ek},$$

(2) The depreciation costs of electric vehicles 

Because the direct government subsidies for purchasing electric vehicles are decreasing, the cost of purchasing electric vehicles is relatively high. Hence, the depreciation costs of electric vehicles in distribution process cannot be neglected. By using the mileage depreciation method, the depreciation costs can be expressed as:

(2) $$C_{2}=\sum_{k\in K}\sum_{i\in V}\sum_{j\in V}x_{ijk}d_{ij}f_{ed},$$

(3) The electricity consumption costs

As recent researches paid little attention to the impact of vehicle load and vehicle speed on energy consumption, the energy consumption of electric vehicles is a fundamental issue in how to dispatch electric vehicles for distribution. Therefore, after reading the relevant literatures about electric vehicle design [27,41], a calculation method of energy consumption considering travel speed and cargo load is presented. In addition, the energy recovery in regenerative braking is also described in the books. Hence, the energy consumption for propulsion is taken into consideration. The power consumption formula is given as:

(3) $$P_{E}=\left[v\left(t\right)/\eta \right]\times \left(Mgf_{r}\cos\alpha +Mg\sin\alpha +0.5\rho_{a}C_{D}A_{f}v{\left(t\right)}^{2}+M\delta \times dv\left(t\right)/dt\right),$$

where $Mgf_{r}\cos\alpha$ is the rolling resistance of tires on the ground, Mgsinα is the grading resistance, $0.5\rho_{a}C_{D}A_{f}v{\left(t\right)}^{2}$ is the aerodynamic drag and Mδ×dv(t)/dt is the acceleration force.

The relevant parameters are shown in Table 2.

Because this paper assumes the distance between each node is Euclidean distance, the road gradient is not taken into consideration and the vehicle speed in the distribution process is constant. That means α and dv(t)/dt are equal to 0. Thus, the power consumption can be simplified as:

(4) $$P_{E}=\left[v/\eta \right]\times \left(Mgf_{r}+0.5\rho_{a}C_{D}A_{f}v^{2}\right),$$

The energy consumption of electric vehicle *k* travelling from note *i* to note *j* can be expressed as:

(5) $$E_{ijk}=x_{ijk}\left[d_{ij}/\left(3600v\right)\right]P_{E}=x_{ijk}\left[d_{ij}/\left(3600\eta \right)\right]\left(W_{ijk}gf_{r}+0.5\rho_{a}C_{D}A_{f}v^{2}\right),\;i,j\in V,\;k\in K,$$

After multiplying the electricity price per kWh, the total electricity consumption costs $C_{3}$ in the distribution can be expressed as follows:

(6) $$C_{3}=\sum_{k\in K}\sum_{i\in V}\sum_{j\in V}c_{e}E_{ijk},$$

(4) The penalty costs for violating customer time window

In the distribution process, the customers generally have a requirement for the expected delivery time. If the cargoes are not delivered to the customer within the time window required. The customer will be unsatisfied. Thus, some penalty costs should be paid. The time when vehicle *k* reaches the customer *i* can be expressed as:

(7) $$T_{i}=\sum_{k\in K}\sum_{j\in V}T_{jik}-ST_{i},\;i\in C,$$

Hence, the penalty costs $C_{4}$ can be calculated as:

(8) $$C_{4}=\sum_{i\in C}\left(w_{ET}max\left\{ET_{i}-T_{i},0\right\}+w_{LT}min\left\{T_{i}-LT_{i},0\right\}\right),$$

(5) The carbon emission trading costs

Although electric vehicles do not emit any carbon emissions during their journey, the power generation in China is mainly based on thermal power plant, accounting for nearly 70 percentages. In addition, according to China’s land transport enterprises greenhouse gas emissions calculation methods released by the government, the electricity consumption needs to be considered when calculating enterprises’ total carbon emissions. Thus, this paper uses Equation (9) to calculate the amount of carbon dioxide emissions from electricity consumption. The indirect carbon emissions EM generated when the vehicle *k* travels between note *i* and note *j* can be expressed as:

(9) $$EM=\alpha_{e}\gamma_{e}E_{ijk},\;i,j\in V,\;k\in K,$$

Based on the research about the impact of carbon trading mechanism on VRP [34,37,39], when the actual carbon emissions are lower the carbon quota allocated, the enterprises can sell the rest quota to those whose actual carbon emissions are higher than the quota to gain profit. In the similar way, if the enterprises emit the carbon emissions exceeding the upper limit, they must purchase additional carbon quota to make up for the excess. Therefore, the carbon trading costs $C_{5}$ can be expressed as:

(10) $$C_{5}=C_{p}\left(\sum_{k\in K}\sum_{i\in V}\sum_{j\in V}\alpha_{e}\gamma_{e}E_{ijk}-Q_{q}\right),$$

On the basis of the above analysis, the MTCCEVRPTW model is expressed as follows:

(11) $$\min F=\;\sum_{k\in K}f_{ek}+\sum_{k\in K}\sum_{i\in V}\sum_{j\in V}\left(x_{ijk}d_{ij}f_{ed}+c_{e}E_{ijk}\right)\;+\sum_{i\in C}\left(w_{ET}max\left\{ET_{i}-T_{i},0\right\}+w_{LT}min\left\{T_{i}-LT_{i},0\right\}\right)\;+C_{p}\left(\sum_{k\in K}\sum_{i\in V}\sum_{j\in V}\alpha_{e}\gamma_{e}E_{ijk}-Q_{q}\right),$$

Subject to:

(12) $$\sum_{j\in V}\sum_{k\in K}x_{ijk}=\sum_{j\in V}\sum_{k\in K}x_{jik}=1,\forall i\in C,$$

(13) $$\sum_{i\in V}x_{ijk}=\sum_{i\in V}x_{jik},\forall j\in \frac{V}{C},\forall k\in K,$$

(14) $$\sum_{i\in V}\sum_{j\in C}x_{ijk}q_{j}\leq W_{max},\forall k\in K,$$

(15) $$\sum_{i\in V}\left(W_{ijk}-W_{jik}\right)=q_{j},\forall j\in C,\;\forall k\in K,$$

(16) $$\sum_{k\in K}W_{max}\geq \sum_{i\in C}q_{i},$$

(17) $$\sum_{i\in V}Q_{ijk}^{out}=Q_{max},\forall j\in \frac{V}{C},\forall k\in K,$$

(18) $$\sum_{j\in V}\left(Q_{jik}^{out}-Q_{ijk}^{in}-E_{ijk}\right)=0,\forall i\in C,\forall k\in K,$$

(19) $$\sum_{i\in V}Q_{ijk}^{out}\geq 0,\forall j\in V,\forall k\in K,$$

(20) $$T_{ijk}=\left(\sum_{j\in V}T_{jik}+d_{ij}/v+ST_{j}\right)\times x_{ijk},\forall i\in V,\forall j\in C,\forall k\in K,$$

(21) $$T_{ijk}=\left(\sum_{j\in V}T_{jik}+d_{ij}/v+CT_{j}\right)\times x_{ijk},\forall i\in V,\forall j\in V/C,\forall k\in K,$$

(22) $$\sum_{i\in V}\sum_{j\in V}\left(d_{ij}\times x_{ijk}\right)\leq d_{max},\forall k\in K,$$

The objective function of the model is to minimize the total costs shown in Equation (11). Equation (12) indicates that each customer must be served once by a vehicle. Equation (13) ensures that the number of entering the charging station equals the number of leaving the charging station. These two equations also indicates that the electric vehicles must return to the distribution center when the distribution tasks are completed. Equation (14) represents that the total load on each path do not pass the maximum load of vehicle. Equation (15) indicates that the load of electric vehicles will be reduced correspondingly after leaving the customer note. Equation (16) imposes the minimum number of electric vehicles for completing the distribution tasks. Equation (17) ensures the battery is full when leaving from the distribution center or charging stations. Equations (18) and (19) indicate the electric vehicles’ energy consumption and it would not break down in the distribution process. Equations (20) and (21) ensure the continuity of the travel time of electric vehicles. Equation (22) ensures the length of each route do not exceed the mileage limit of electric vehicle per day.

### 3.4. CVRPTW Model

Based on the recent researches about traditional fuel vehicle routing problem combining carbon trading [7,35,36,37,38,39,40], this paper defines the sub-costs and constructs the CVRPTW model as follows.

(1) The fixed costs of fuel vehicles

(23) $$C_{1}=\sum_{k\in K}f_{fk},$$

(2) The depreciation costs of fuel vehicles

(24) $$C_{2}=\sum_{k\in K}\sum_{i\in V}\sum_{j\in V}x_{ijk}d_{ij}f_{fd},$$

(3) The fuel consumption costs

There are some scholars have come up with a linear function for fuel consumption [37,39]. The linear function is presented as follows:

(25) $$\rho \left(X\right)=\rho_{0}+\frac{\rho^{*}-\rho_{0}}{W_{max}}X,$$

Hence, the fuel consumption of vehicle *k* travelling form note *i* to note *j* can be expressed as:

(26) $$E_{ijk}=x_{ijk}d_{ij}\rho \left(W_{ijk}\right),$$

The fuel consumption costs $C_{3}$ are expressed as:

(27) $$C_{3}=\sum_{k\in K}\sum_{i\in V}\sum_{j\in V}c_{f}E_{ijk},$$

(4) The penalty costs

The time when vehicle reaches the customer *i* is $T_{i}$, thus the penalty costs can be expressed as:

(28) $$C_{4}=\sum_{i\in C}\left(w_{ET}max\left\{ET_{i}-T_{i},0\right\}+w_{LT}min\left\{T_{i}-LT_{i},0\right\}\right),$$

(5) The carbon trading costs

Different with carbon emissions from using electricity, the fuel vehicles will emit carbon dioxide in the distribution process directly. This paper gives the expression as: $Carbon\;emissions\;=\;fuel\;consumption\times CO_{2}\;emission\;factor$. There is fuel emission factor, such as gasoline and diesel, which is different with the grid emission factor mentioned above.

The fuel consumption EM of vehicle *k* travelling from note *i* to note *j* can be expressed as:

(29) $$EM=\gamma_{f}E_{ijk},$$

Hence, the carbon trading costs $C_{5}$ is expressed as:

(30) $$C_{5}=C_{p}\left(\sum_{k\in K}\sum_{i\in V}\sum_{j\in V}\gamma_{f}E_{ijk}-Q_{q}\right),$$

Based on the analysis above, the MTCCVRPTW model is constructed as follows:

(31) $$\min F=\;\sum_{k\in K}f_{fk}+\sum_{k\in K}\sum_{i\in V}\sum_{j\in V}\left(x_{ijk}d_{ij}f_{fd}+c_{f}E_{ijk}\right)\;+\sum_{i\in C}\left(w_{ET}max\left\{ET_{i}-T_{i},0\right\}+w_{LT}min\left\{T_{i}-LT_{i},0\right\}\right)\;+C_{p}\left(\sum_{k\in K}\sum_{i\in V}\sum_{j\in V}\gamma_{f}E_{ijk}-Q_{q}\right),$$

Subject to:

(32) $$\sum_{i\in V}x_{i0k}=\sum_{i\in V}x_{oik}=1,\forall k\in K,$$

(33) $$\sum_{k\in K}\sum_{i\in V}x_{ijk}=\sum_{k\in K}\sum_{i\in V}x_{jik}=1,\forall j\in C,$$

(34) $$\sum_{i\in V}\sum_{j\in C}x_{ijk}q_{j}\leq W_{max},\forall k\in K,$$

(35) $$\sum_{i\in V}\left(W_{ijk}-W_{jik}\right)=q_{j},\forall j\in C,\;\forall k\in K,$$

(36) $$\sum_{k\in K}W_{max}\geq \sum_{i\in C}q_{i},$$

(37) $$T_{j}=\sum_{k\in K}\sum_{i\in V}x_{ijk}\left(T_{i}+d_{ij}/v+ST_{i}\right),\forall j\in C,$$

(38) $$\sum_{i\in V}\sum_{j\in V}\left(d_{ij}\times x_{ijk}\right)\leq d_{max},\forall k\in K,$$

Equation (32) presents that all the vehicles must return to the distribution center when the task is completed. Equation (33) indicates that each customer is only be visited once. Equation (34) shows that the vehicle cannot be overload. Equation (35) indicates that the load of electric vehicles will be reduced correspondingly after leaving the customer note. Equation (36) shows the minimum number of vehicles for completing the distribution tasks. Equation (37) indicates the service continuity for two customer nodes. Equation (38) ensures the length of each route do not exceed the mileage limit of vehicles per day.

## 4. Model Solution

**Figure 2:**
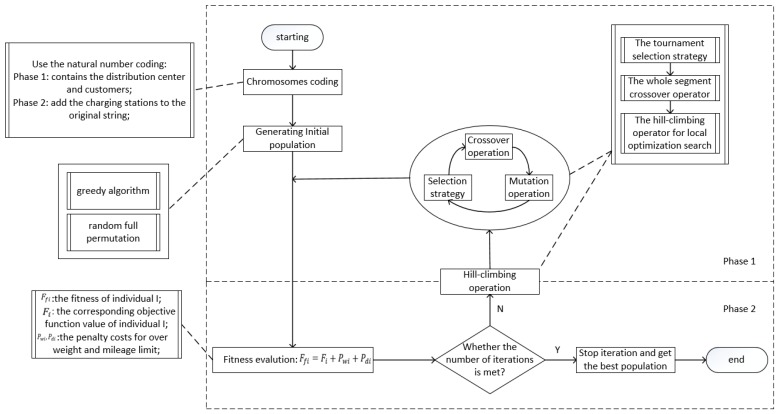
Basic process of hybrid genetic algorithm (HGA).

Since the traditional VRP is NP-hard problem, EVRP is also NP-hard problem. This paper uses a HGA to solve these two problems. However, genetic algorithm has good global search ability and scalability, but its local search ability is insufficient. Thus, the traditional selection operator is difficult to ensure the diversity of the population. In particular, when solving the problem of more constraints such as EVRP, the traditional crossover operator tends to destroy the personality of good individuals, resulting in low efficiency. Therefore, this paper proposes the algorithm improvement to cover the above shortcomings. Firstly, a greedy algorithm is designed to improve the initial population quality. Then in order to keep the good personality of the father to the children as much as possible, the crossover operators are designed by a whole segment exchange method for the complex constraints of EVRP. Finally, the hill-climbing operator is embedded to optimize the neighborhood of the excellent individual, which is beneficial to improve the convergence and accuracy of the algorithm. Its basic process is illustrated in Figure 2.

### 4.1. Coding

While solving EVRP, as the assumption that the electric vehicles are not allowed to charge by their own, a two-phase coding strategy is adopted (for VRP, only the first phase is conducted due to ignoring the refueling stations). In the first phase, the traditional natural number coding for VRP with time windows is adopted, excluding the charging stations. The crossover and other operations is also completed by this coding way. In the second phase, when calculating the fitness of each individual, the charging stations are added to the scheme reflected by the code string according to the electricity consumption. In the third phase, the hill-climbing and selection operation is obtained according to fitness ordering, so as to do the neighborhood optimization and save the good individuals for next generation. These operations can help to solve EVRP and VRP more efficiently.

### 4.2. Producing Feasible Initial Population

A greedy algorithm and random full permutation are adopted to generate initial population. The individuals generated by using greedy algorithm have good performance, on the other hand, half of the rest are generated by random full permutation, which can increase population diversity. The main principle of greedy algorithm is an insertion algorithm based on a time series. When allocating the customers to vehicles, the aim of minimum penalty cost is set. If the electric or fuel vehicles would arrive the customer in time, this customer node is inserted to the corresponding sub-string. If all the vehicles could not arrive in time, the minimum penalty cost is chosen correspondingly.

### 4.3. Determining Fitness Function and Fitness Calculation

Before calculating the fitness value, the charging stations node is added to the coding string. First the electricity consumption is calculated according to Equation (5), ensuring the electric vehicles will not be out of power half the way. Then, the nearest charging station node is inserted to the original coding string. Finally, the fitness value of each individual is calculated. The fitness value equals the objective function value plus the penalty costs for over load and mileage limit. Besides, because the tournament selection strategy is taken, there is no need to take the reciprocal of objective function. Thus, the fitness function is expressed as:

(39) $$F_{fi}=F_{i}+P_{wi}+P_{di},$$

where $F_{fi}$ represents the fitness value of individual *i*, and $F_{i}$ represents the objective function value of individual *i*. $P_{wi}$ and $P_{di}$ represent the penalty costs for over load and mileage limit of individual *i*. In details, it can be expressed as:

(40) $$F_{fi}=F_{i}+M_{1}\sum_{k\in K}\sum_{i\in V}\sum_{j\in V}max\left(W_{ijk}-W_{max},0\right)\;+M_{2}\sum_{k\in K}max\left(\sum_{i\in V}\sum_{j\in V}d_{ij}x_{ijk}-d_{max},0\right),$$

where $M_{1}$ and $M_{2}$ are a maximum positive value.

### 4.4. Selection Operation

This paper uses the tournament selection to conduct selection operation, which can ensure the better individual selected with higher probability and remove the inferior individuals. Hence, the convergence speed could be increased.

### 4.5. Crossover Operation

**Figure 3:**
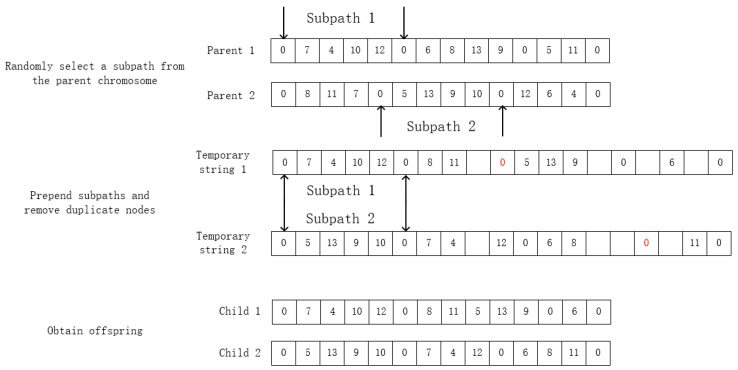
Description of crossover operation example.

Due to the drawbacks of traditional crossover operators while solving the multiple constraints routing model, this paper uses a whole segment crossover operators to conduct crossover operation, which could retain the personality of better individuals to greatest extent. The detailed information is shown in Figure 3.

### 4.6. Mutation Operation

This paper adopts the interchange mutation strategy to conduct the mutation operation.

### 4.7. Hill-Climbing Operation

Because the hill-climbing algorithm is a search algorithm with good local optimization ability but weak in global optimization, this paper conducts the hill-climbing algorithm to best individual in each generation. Two exchange points in one sub-path of the coding string are randomly selected, which can ensure exchanging the customers nodes will not result in overloading. This could help to improve the HGA efficiency.

### 4.8. Generating a New Generation Population

A new population could be generated based on Step (4–7).

### 4.9. Terminating Condition

The termination condition is whether the current number of iterations is greater than maximum number of iterations. If the condition is met, the loop would break, otherwise, the loop continues.

### 4.10. Decoding

By adding the charging stations nodes to the coding string of best solution, the actual operational plan can be obtained.

Since the parameter setting for HGA has a great influence on the algorithm’s ability to solve the problem, the quality and efficiency of the results would be affected. According to a large number of experiments, the parameters are set as follows: the number of generations is 3000, the crossover probability is 0.9, the mutation probability is 0.05, the initial population is 100, and the number of hill-climbing in each generation is 100.

## 5. Experimental Design and Results

### 5.1. Experimental Design

Firstly, the experimental design is used to verify the effectiveness of CEVRPTW and CVRPTW model, and then compare the experiments results to explore the impact of adopting electric vehicles for distribution on total costs and carbon emissions. Secondly, for CEVRPTW, the impact of changes of battery capacity and charge rate on the total costs and carbon emissions is discussed. Thirdly, the impact of changes of carbon price and quota on the distribution scheme and total costs of electric and fuel vehicles is studied respectively and the comparative analysis is provided. 

The case chosen as below is selected from EVRP data set rc107_21 provided by [19], which made appropriate modifications based on the Solomon data set, which is well-known in VRPTW. Besides this, an appropriate adjustment for this case is given according to the coordinate information of distribution center, charging stations and customers selected from EVRP data set rc107_21. The demand of customer takes values from [0,0.2] and the service time of customer takes from [0.1,0.5] randomly. Moreover, at the present stage, the logistics companies usually choose the fast charging model to recharge their electric vehicles in the distribution process for delivering goods to customers in time. According to the data from State Grid Corporation of China (SGCC) and the Society of Automotive Engineers (SAE), the rated power of charging piles for fast charging takes at least 60 kW and the rated voltage is at least 200 v. The charging time (to 100% capacity) could be 0.5–1 h. Therefore, the value of charging time is assumed to be 0.5 h in this paper. The desirable time window of customer takes value from [0,7]. The details of the selected example is shown in Table 3. The values of the constant parameters in Equation (5) are determined by [27,41], which is shown in Table 4. The value of battery capacity is also taken from these two references, which is 27 kWh. According to the experiences from logistics companies, the values of fixed costs, depreciation costs, and other relevant parameters of electric and fuel vehicles are determined, which are shown in Table 5 and Table 6 respectively. Besides, the values of unit electricity price and fuel price are taken from the Chongqing commercial electricity price and gasoline price in May 2019. The value of proportion of thermal power generation is taken according to the data provided by State Statistical Bureau and the emission factor of grid and fuel are determined by the data from National Climate Centre and Intergovernmental Panel on Climate Change (IPCC) Guidelines for National Greenhouse Gas Inventories respectively. The maximum load $W_{max}$ for electric vehicle and fuel vehicle are both 1t. The vehicle speed goes to 40 km/h, the penalty costs of unit time $w_{ET}$ and $w_{LT}$ for arriving the customer earlier or late are 20 and 30 Yuan/h respectively. Lastly, the mileage limits for electric vehicles and fuel vehicles are both 240 km per day.

### 5.2. Experimental Results

**Figure 4:**
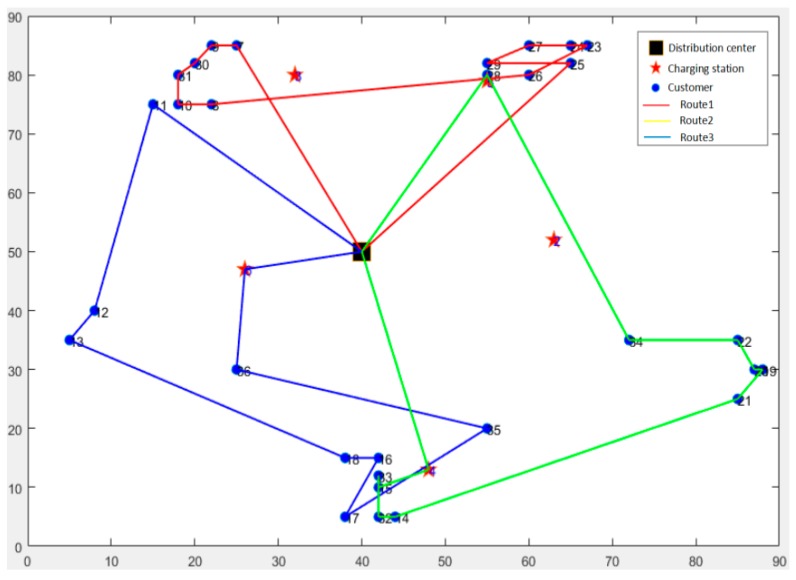
The optimal distribution paths of Capacitated Electric Vehicle Routing Problem with Time Windows (CEVRPTW).

**Figure 5:**
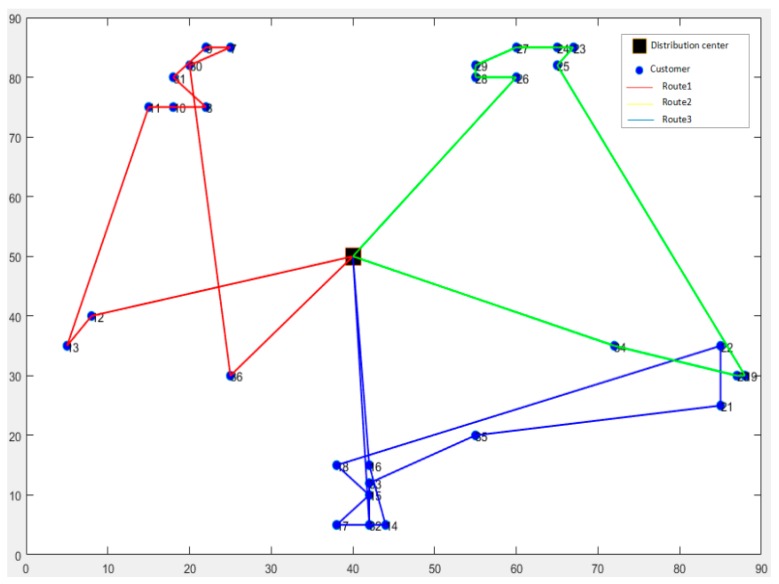
The optimal distribution paths of Capacitated Fuel Vehicle Routing Problem with Time Windows (CVRPTW).

The initial value of carbon price and quota are taken as 0.05 Yuan/kg and 0 respectively and the number of vehicles is set to be 3. The example is used with HGA to solve these two models and each is conducted for 20 times. Then the results with minimum objective value are taken as the optimal solution and path scheme corresponding to these two models. The distribution schemes for CEVRPTW and CVRPTW are shown in Figure 4 and Figure 5. And the comparison with two results is given in Table 7.

Then, sensitivity analysis is given to explore the impact of relevant parameters of electric vehicles on total costs and carbon emissions, shown in Section 5.2.1. In Section 5.2.2, the experiments’ results of CEVRPTW and CVRPTW are discussed when carbon prices and carbon quotas change. Based on these results, four inferences are provided:

Inference I: As the battery capacity and charge rate increase larger, the total costs of electric vehicles will decrease correspondingly, but this download trend will gradually level off. The carbon emissions decrease with the increase of battery capacity, but have little to do with the charge rate.

Inference II: The total costs of CEVPTW have a trend to be lower than that of CVRPTW while the carbon price increases. In this case, when carbon price increases from 0.05 to 1.25 Yuan/kg, the gap amount of total costs decrease from 291.7 to 138.8 Yuan.

Inference III: Both for CEVRPTW and CVRPTW, once carbon price is a fixed value, the optimal distribution paths will not change with the carbon quotas. 

Inference IV: As the carbon price increases, carbon emissions of fuel vehicles will decrease but it has little influence on carbon emissions of CEVRPTW. In this case, when carbon price increases from 0.05 to 1.25 Yuan/kg, the carbon emissions of CVRPTW decreases from 227.6 to 197.2 kg, but that of CEVRPTW fluctuate around 37 kg.

From the results in Table 7, the following findings can be obtained:

(1) The carbon emissions of CEVRPTW is quite lower than that of CVRPTW. Thus, using electric vehicles in the distribution process can greatly reduce carbon emissions in logistics enterprises.
(2) The solution of CEVRPTW has a higher penalty costs, which means using electric vehicles results in a low customer satisfaction. Also, as the battery capacity determines the maximum cruising range and the charge rate influences the stay time at charging stations. Thus, in Section 5.2.1, the sensitivity analysis about the battery capacity and charge rate is given.
(3) The carbon trading costs for CEVRPTW and CVRPTW are relatively small, accounting for less than 1% of the total costs when the carbon price is 0.05 Yuan/kg, which are 1.86 Yuan and 11.4 Yuan respectively. However, the gap amount of carbon emissions is comparatively large, which is 190.3 kg. Thus, in Section 5.2.2, the impact of carbon price and carbon quota on CVRPTW and CEVRPTW is analyzed respectively.

#### 5.2.1. Sensitivity Analysis

**Figure 6:**
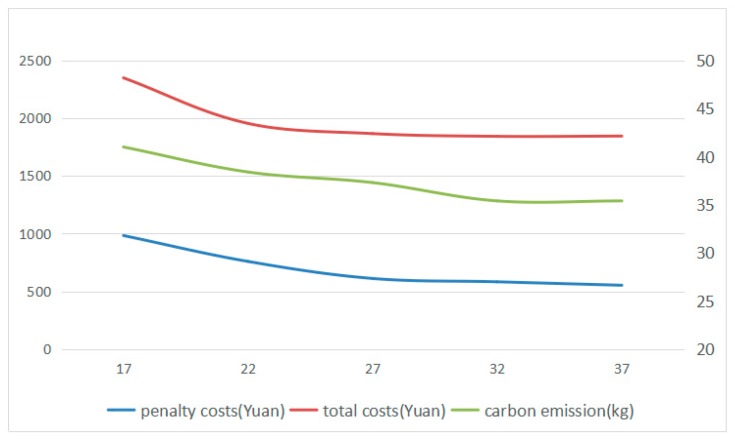
The change of penalty costs, total costs, and carbon emission under different battery capacity.

In order to prove Inference I, the initial value of the battery capacity $Q_{max}$ is taken as the center point and the interval is 5 kWh, which can present the relationship between battery capacity and total costs and carbon emissions more clearly. Then, $Q_{max}$ is set to be 17, 22, 27, 32, 37 kWh for 5 groups of experiments and each group is solved by 20 times, and the optimal solution that has optimal fitness value in each group can be selected. The results are shown in Figure 6.

**Figure 7:**
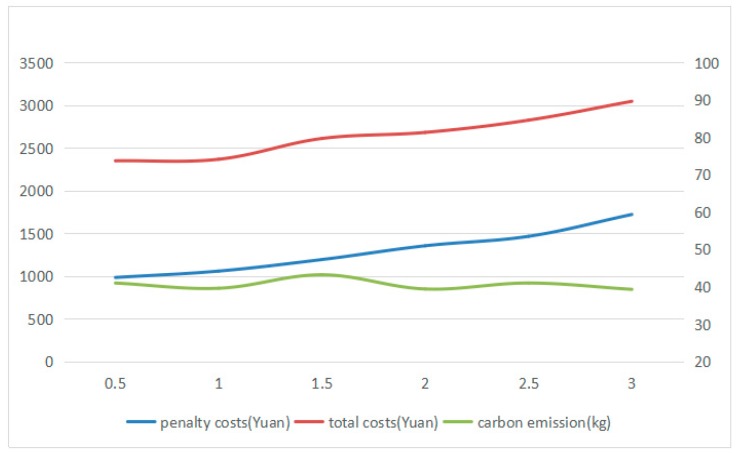
The change of penalty costs, total costs, and carbon emission under different charging time.

Since the logistics companies usually recharge their electric vehicles in regular charging or fast charging mode in the distribution process, the charging time (to 100% capacity) in regular charging model is 2–3 h generally. The maximum rated power is 10 kW. Therefore, the charging time of electric vehicles CT is set to be 0.5 h, 1 h, 1.5 h, 2 h, 2.5 h, and 3 h for 6 groups of experiments to reflect the charge rate of electric vehicles. In addition, in order to present the relationship between charge rate and total costs and carbon emissions more clearly, the battery capacity is set to be 17 kWh for the vehicles that would be charged more frequently. In the same way, the experiments for 20 times are conducted and the optimal solution is selected. And the results are shown in Figure 7.

From Figure 6, it can be found that when battery capacity goes up from 17 kWh to 32 kWh, the penalty costs, total costs, and carbon emissions gradually decrease, which confirmed part of Inference I. When battery capacity continues to go up, the penalty cost, total cost, and carbon emissions tend to be gentle. 

From Figure 7, it can be obtained that the penalty costs and total costs increased steadily while the charging time goes up from 0.5 h to 3 h. However, the value of carbon emissions is fluctuating around 38 kg. Thus, the Inference I is proved.

Both battery capacity and charge rate have a great importance on the total costs of using electric vehicles for distribution. Low battery capacity will result in the increase of frequency in charging, and low charge rate will lead to the long waiting time at the charging station, which may cause the electric vehicles violate the time window that the customers demand. And from the perspective of carbon emissions, the charge rate has little thing to do with carbon emissions but battery capacity influences a lot, which might help the logistics companies selecting the model of electric vehicles.

#### 5.2.2. Carbon Price and Quota Analysis

In carbon trading situations, the company which emits less carbon dioxide than its quota can sell the rest quota to other companies for profit. And adopting the electric vehicles for distribution can effectively reduce carbon emissions, which means the enterprises those adopt electric vehicles can make profit from carbon trading market. Besides, the carbon price has been proved as a determinative role in carbon trading [42,43], which may indirectly change vehicle arrangements and route planning [37,39]. In the following. A detailed study of CEVRPTW and CVRPTW on carbon prices and carbon quotas is conducted respectively.

For Inference II, the carbon prices $C_{p}$ is set to be 0.05, 0.25, 1.25 Yuan/kg and carbon quota is set to be 30, 90, 270 kg. Each group of data is taken into the model to solve 20 times and the best solution is selected. The results are shown in Table 8.

According to Table 8, the following findings can be observed:

(1) Once the carbon price is a certain value, the carbon emissions do not change whether the carbon quota changes or not, which means the distribution paths will not change with the carbon quota. This finding support Inference III, and its mathematical process is presented as below. The total costs of CEVRPTW F is expressed as:

(41) $$\min F=C_{1}+C_{2}+C_{3}+C_{4}+C_{p}\left(\sum_{k\in K}\sum_{i\in V}\sum_{j\in V}\alpha_{e}\gamma_{e}E_{ijk}-Q_{q}\right),$$

Once $C_{p}$ and other parameters do not change, the other sub-cost $C_{1},C_{2},C_{3},C_{4}$ will not change apparently. The change of carbon quota is assumed as $Q_{change}$, the total costs of CEVRPTW F can be expressed as:

(42) $$\min F_{1}=C_{1}+C_{2}+C_{3}+C_{4}+C_{p}\left(\sum_{k\in K}\sum_{i\in V}\sum_{j\in V}\alpha_{e}\gamma_{e}E_{ijk}-(Q_{q}+Q_{change})\right)\;=C_{1}+C_{2}+C_{3}+C_{4}+C_{p}(\sum_{k\in K}\sum_{i\in V}\sum_{j\in V}\alpha_{e}\gamma_{e}E_{ijk}-Q_{q})-C_{p}Q_{change}\;=\min F-C_{p}Q_{change},$$

Because $C_{p}$ and $Q_{change}$ are fixed values, $C_{p}Q_{change}$ equals a constant, named A. Hence, $F_{1}$ can be expressed as:

(43) $$\min F_{1}=minF-A,$$

Obviously, the change of carbon quota has no impact on carbon emissions and distribution paths of CEVRPTW. In similar way, the change of carbon quota has no impact on carbon emissions and distribution paths of CVRPTW. The Inference III has been proved. And according to Equation (42), it can be found that the total costs will change accordingly.

(2) While the carbon price $C_{p}$ is 0.05, 0.25, 1.25 Yuan/kg, the carbon emissions of CEVRPTW are 37.3, 38.2 and 36.1 kg, and the carbon emissions of CVRPTW is 227.6, 216.8 and 197.2kg, presenting a downward trend, which has supported Inference IV. The proportion of fixed costs of vehicles $C_{1}$, depreciation costs $C_{2}$, energy costs $C_{3}$, penalty costs $C_{4}$, and carbon trading costs $C_{5}$ are analyzed in the total cost of distribution F when $C_{p}$ is set to be 0.05, 0.25, and 1.25 Yuan/kg and $Q_{q}$ is set to be 0 kg. The cost proportion analysis is shown in Table 9, Table 10 and Table 11.

**Figure 8:**
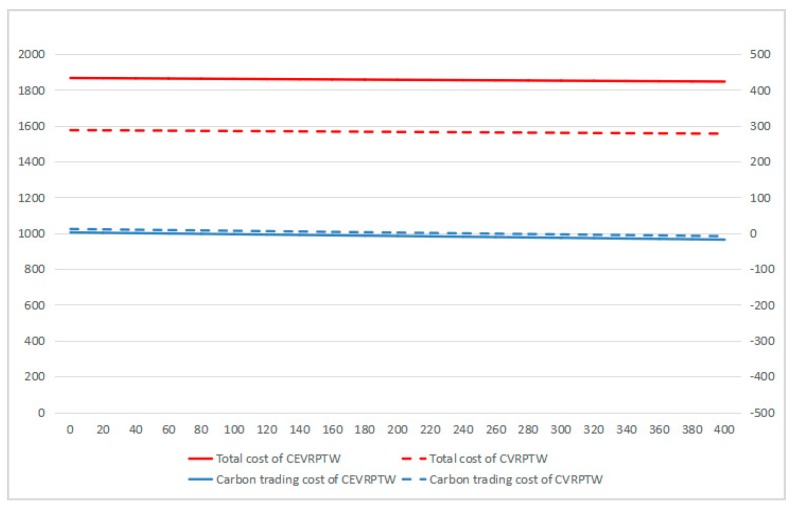
The change of carbon trading cost, total cost under different carbon quota when carbon price is 0.05.

**Figure 9:**
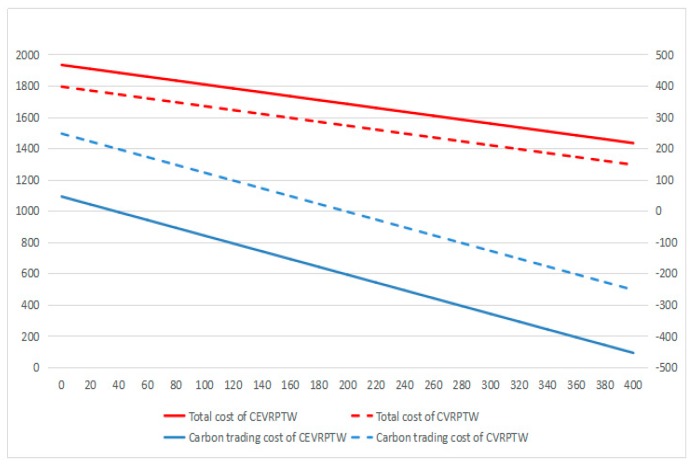
The change of carbon trading cost, total cost under different carbon quota when carbon price is 1.25.

**Figure 10:**
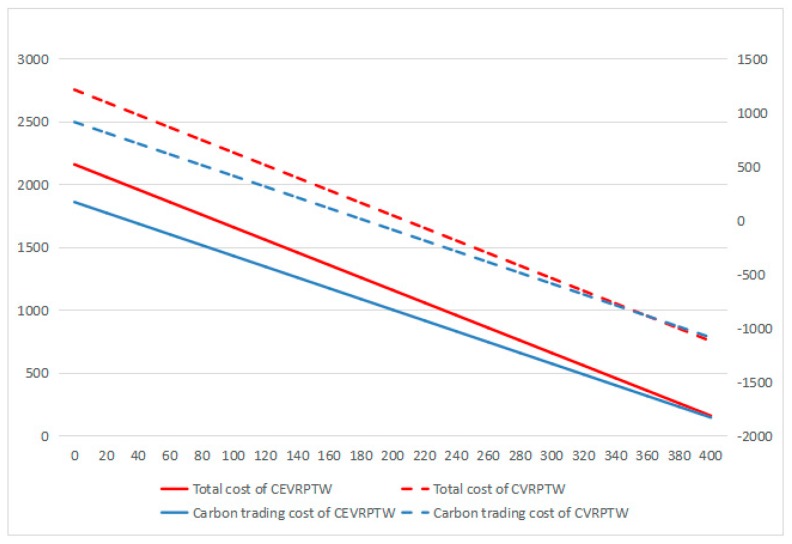
The change of carbon trading cost, total cost under different carbon quota when carbon price is 5.

According to Figure 8, Figure 9 and Figure 10, it can be found that for CEVRPTW, the proportion of carbon trading costs $C_{5}$ is too low, for only accounting for 2.34% even when carbon price is 1.25 Yuan/kg. Thus, the carbon trading costs has no significant effect on the total cost of distribution and distribution scheme.

In contrast, the proportion of carbon trading costs $C_{5}$ of CVRPTW is much higher than that of CEVRPTW, when the carbon price is 1.25 Yuan/kg, the proportion is 13.75%. Therefore, the result supports Inference IV that carbon emissions of CVRPTW will decrease when carbon price increases and the impact of carbon price on carbon emissions of CEVRPTW is relatively less.

(3) When carbon price is 0.05, 0.25, 1.25 Yuan/kg, the gap of total costs of CEVRPTW and CVRPTW is gradually narrowing. The gap is 291.3 Yuan when the carbon price is 0.05 Yuan/kg. The gap decreases to 203.1 Yuan when carbon price is 0.25 Yuan/kg. Finally the gap decreases to 138.8 Yuan, which has supports Inference II.

Finally, the carbon quota is set to be 20 groups from 20, 40, …, 400 kg and the value of carbon price is taken as 0.05 (comparatively small), 1.25 (medium), and 5 (comparatively large) Yuan/kg, so as to present the relationship of carbon quota and gap of total costs of CEVRPTW and CVRPTW.

As shown in Figure 8, Figure 9 and Figure 10, while the carbon price is comparatively small, for only 0.05 Yuan/kg, it can be seen that the total costs of CEVRPTW and CVRPTW almost do not change. While the carbon price goes up to 1.25, the total costs of CEVRPTW chase the total costs of CVRPTW and the gap is only 203.1 Yuan. The costs of CEVRPTW finally surpass the CVRPTW when the carbon price is 5 Yuan/kg, which means this paper adopting electric vehicles is more economical than using fuel vehicles in such a situation. When the carbon quota increases, the total costs and carbon trading cost decreases steadily no matter what the carbon price is, which means carbon quota has no influence on the transformation of logistics enterprises.

In summary, if a logistics enterprise wants to decide whether to adopt electric vehicles from a totally economical view, it needs to take into account the carbon price instead of the carbon quota.

## 6. Conclusions and Future Works

With high energy consumption and environmental pollution problems becoming more and more serious, it is an inevitable trend for the land transportation companies to adopt new energy vehicles. China government has also published a set of relevant policies to promote the new energy vehicles’ development. Although there has been a large subsidy for purchasing in the past few years, the direct subsidy is decreasing year by year, slowly replaced by carbon trading, which is a more market-oriented means. It has achieved good results and been proved as an effective way to reduce carbon emissions. On the other hand, although logistics companies have been included in carbon trading market in some pilot cities, the coverage is not large, only the airline and shipping companies are included. However, a large proportion of carbon emissions are emitted on the land, which cannot be neglected. It needs some time for carbon trading to develop to involve the land transportation companies. Thus, under the new energy background, this paper firstly proposes a combinatorial optimization of electric vehicle routing problem, then sensitivity analysis about battery capacity and charge rate is given. Secondly, in order to explore the difference of the costs by using electric vehicles or fuel vehicles of land transportation companies under carbon trading market, a CVRPTW model is established and solved by HGA to conduct numerical experiments both for CEVRPTW and CVRPTW under different carbon price and quota.

The results of this paper are concluded as follows: (1) adopting electric vehicles for distribution can indeed reduce the carbon emissions; (2) the battery capacity and charge rate of electric vehicles have a great impact on the total costs of distribution process; as the battery capacity goes up, the carbon emissions will decrease and finally be close to a certain value; (3) when carbon price is a fixed value, the change of carbon quotas cannot cause changes of carbon emissions both for CEVRPTW and CVRPTW, but can cause changes in total costs and carbon trading costs; (4) with the increasing carbon price, the carbon emissions of CVRPTW will fall steadily, but it has no significant impact on the carbon emissions of CEVRPTW; (5) carbon price plays a vital role in the transformation of land transportation companies. When the carbon price is a relatively small value, the increase of carbon quota has no obvious impact on the total costs of CERVPTW and CVRPW. However, when the carbon price rises, the gap of total costs narrows, and finally the total costs of CEVRPTWare lower than that of CVRPTW, which means the logistics companies are willing to adopt electric vehicles to complete the distribution task instead of the fuel vehicles. These conclusions are expected to provide decision support for the government and the managers of land transportation enterprises. For the government, with the national carbon trading market developing gradually, how to make policies to influence the market carbon price to encourage the popularity of electric vehicles is vital. For the enterprises, it can support to build transportation strategies for logistics management under carbon trading market.

This paper studies an optimization EVRP and compares the distribution costs by using electric vehicles and fuel vehicles under carbon trading. It firstly considers the indirect carbon emissions of electric vehicles and compares with fuel vehicles under carbon trading in China. However, on the one hand, there will be a more complex environment in actual operation of distribution, such as real road network, the traffic jam in the road and etc. In future research, the real geographical situations and the road congestion could be considered. And the electric vehicles charging model could also be considered, for example, choosing battery swapping or charging to full or half full would have an impact on the distribution process. In addition, future research could combine the advantages of electric vehicles and fuel vehicles and investigate the optimization of mixed fleet vehicles, such as adopting fuel vehicle to complete the transportation from the countryside to city and then using electric vehicles to complete the “last mile” distribution task.

## Figures and Tables

**Table 1:** Description of symbols.

C	set of customers (C={s+1,s+2…,s+c})
S	set of charging stations (S={1,2…,s})
O	distribution center (O={0})
V	collection of all nodes, V=C∪S∪O
K	set of vehicles actually used
$d_{ij}$	the distance between note *i* and *j*, i,j∈V
v	the vehicle travel speed
$\alpha_{e}$	the proportion of thermal power generation
$\gamma_{e}$	the grid emission factor
$\gamma_{f}$	the fuel emission factor
$C_{p}$	the unit carbon price
$Q_{q}$	amount of carbon emissions quota which is allocated by government
$\left[ET_{i},LT_{i}\right]$	the time window that customer *i* expected to be satisfied, i∈C
$w_{ET}$	the penalty costs for unit time when the vehicle arrives at the customer in advance
$w_{LT}$	the waiting costs of customer for unit time when the vehicle arrives late
$ST_{i}$	the time required for vehicle to serve customer *i*, i∈C
$CT_{i}$	the charging time in charging station *i*, i∈S
$T_{ijk}$	the departing time of vehicle *k* from note *j* after traveling from note *i* to *j*, i,j∈V
$f_{ed}$	the depreciation cost of electric vehicles per kilometer
$f_{fd}$	the depreciation cost of fuel vehicles per kilometer
$f_{ek}$	the fixed cost of electric vehicles
$f_{fk}$	the fixed cost of fuel vehicles
$c_{e}$	the unit electricity price
$c_{f}$	the unit fuel price
ρ	the fuel consumption per unit distance when the vehicle is running
$\rho_{0}$	the fuel consumption per unit distance when the vehicle is empty
$\rho^{*}$	the fuel consumption per unit distance when the vehicle is fully loaded
$q_{i}$	the demand of customer *i*, i∈C
$E_{ijk}$	the energy consumption of vehicle *k* between travelling from note *i* to note *j*, i,j∈V
$W_{max}$	the maximum load allowed for vehicles
$W_{ijk}$	the weight of vehicle when it travels between note *i* to *j*, i,j∈V
$Q_{ijk}^{in}$	the rest amount of electricity after travelling from note *i* to note *j*, i,j∈V
$Q_{ijk}^{out}$	the amount of electricity when departing from note *j* after travelling from note *i* to *j*, i,j∈V
$Q_{max}$	the maximum battery capacity
$d_{max}$	the maximum mileage per day
$x_{ijk}$	if the vehicle *k* travels note *j* from note *i*, $x_{ijk}$ is 1, otherwise $x_{ijk}$ is 0

**Table 2:** The description of symbols in formula (3).

$P_{E}$	battery power output (W)
v(t)	the vehicle travel speed (m/s)
η	efficiency parameter to account for any power losses in the transmission and the motor drive (dimensionless)
M	vehicle mass (kg)
g	gravitational constant (m/s^2^)
$f_{r}$	rolling resistance coefficient (dimensionless)
α	angle of the road (−(−π/2)≤α≤(π/2), in radians)
$\rho_{a}$	air density (kg/m^3^)
$C_{D}$	aerodynamic drag coefficient (dimensionless)
$A_{f}$	vehicle frontal area (m^2^)
δ	mass factor (dimensionless)
dv(t)/dt	Acceleration (m/s^2^)

**Table 3:** Information of the selected example.

Number	Note	Coordinates (km)		Demand (t)	Service Time (h)	Desirable Time (h)	
		x	y			$ET_{i}$	$LT_{i}$
0	Distribution center	40.0	50.0	0	0	0.0	16
1	Charging station1	63.0	52.0	0	0.5	0.0	16
2	Charging station2	32.0	80.0	0	0.5	0.0	16
3	Charging station3	48.0	13.0	0	0.5	0.0	16
4	Charging station4	55.0	79.0	0	0.5	0.0	16
5	Charging station5	26.0	47.0	0	0.5	0.0	16
6	Customer1	25.0	85.0	0.1	0.5	0.0	1
7	Customer2	22.0	75.0	0.05	0.5	0.5	2.5
8	Customer3	22.0	85.0	0.05	0.5	1	3
9	Customer4	18.0	75.0	0.1	0.3	1	3
10	Customer5	15.0	75.0	0.1	0.1	2	3
11	Customer6	8.0	40.0	0.2	0.5	0.0	1
12	Customer7	5.0	35.0	0.05	0.2	1	4
13	Customer8	44.0	5.0	0.1	0.3	1	2
14	Customer9	42.0	10.0	0.2	0.1	2	5
15	Customer10	42.0	15.0	0.05	0.5	0.5	2
16	Customer11	38.0	5.0	0.05	0.5	2	4
17	Customer12	38.0	15.0	0.1	0.4	1.5	3
18	Customer13	88.0	30.0	0.1	0.3	1.5	4.5
19	Customer14	87.0	30.0	0.15	0.2	1	2
20	Customer15	85.0	25.0	0.05	0.5	3	5
21	Customer16	85.0	35.0	0.1	0.1	3	5
22	Customer17	67.0	85.0	0.1	0.5	3	5.5
23	Customer18	65.0	85.0	0.05	0.5	2.5	5.5
24	Customer19	65.0	82.0	0.1	0.4	3.5	5.5
25	Customer20	60.0	80.0	0.05	0.3	4	6
26	Customer21	60.0	85.0	0.1	0.2	4.5	5
27	Customer22	55.0	80.0	0.1	0.5	0.0	1
28	Customer23	55.0	82.0	0.05	0.4	3.5	5.5
29	Customer24	20.0	82.0	0.2	0.3	4	5
30	Customer25	18.0	80.0	0.05	0.1	1	5
31	Customer26	42.0	5.0	0.1	0.5	3	6
32	Customer27	42.0	12.0	0.05	0.3	4.5	6.5
33	Customer28	72.0	35.0	0.05	0.4	0.5	2
34	Customer29	55.0	20.0	0.2	0.5	4.5	6.5
35	Customer30	25.0	30.0	0.05	0.2	5	7

**Table 4:** Values of the constant parameters in the energy consumption formula.

Constant Parameter	Value
η	0.8
g	9.8 m/s^2^
$f_{r}$	0.01
$\rho_{a}$	1.205 kg/m^3^
$C_{D}$	0.6
$A_{f}$	3.504 m^2^
δ	1.1
M	1800 kg

**Table 5:** Parameters of Capacitated Electric Vehicle Routing Problem with Time Windows (CEVRPTW) model.

Parameter	Value
$\;f_{ek}$	100 Yuan
$f_{ed}$	1.5 Yuan/km
$c_{e}$	0.82 Yuan/kWh
$\alpha_{e}$	0.73
$\gamma_{e}$	0.65 kg/kWh

**Table 6:** Relevant parameters of Capacitated Fuel Vehicle Routing Problem with Time Windows (CVRPTW) model.

Parameter	Value
$f_{fk}$	100 Yuan
$f_{fd}$	0.33 Yuan/km
$c_{f}$	7.25 Yuan/L
$\gamma_{f}$	2.63 kg/L
$\rho_{0}$	0.10 L/km
$\rho^{*}$	0.21 L/km

**Table 7:** Comparison of results for CEVRPTW and CVRPTW.

Problem Type	CEVRPTW	CVRPTW
Number of vehicles	3	3
Fixed costs (Yuan)	300	300
Travel distance (km)	590.8	577.8
Depreciation costs (Yuan)	886.2	190.7
Penalty costs (Yuan)	612.9	444.7
Energy consumption costs (Yuan)	64.5	627.5
Carbon trading costs (Yuan)	1.86	11.4
Carbon emissions (kg)	37.3	227.6
Total costs (Yuan)	1865.6	1574.3

**Table 8:** The results of CEVRPTW and CVRPTW with different carbon price and quota.

Type	$C_{p}$	$Q_{q}\;(\mathrm{kg})$	Total Costs	Carbon Costs	Carbon Emissions (CE)	$Q_{q}-CE$
CEVRPTW	0.05	30	1864.5	0.37	37.3	−7.3
		90	1861.5	−2.65	37.3	52.7
		270	1852.5	−11.64	37.3	232.7
	0.25	30	1866.8	2.05	38.2	−8.2
		90	1851.8	−12.95	38.2	51.8
		270	1793.9	−57.9	38.2	231.8
	1.25	30	1894.8	−7.6	36.1	−6.1
		90	1819.8	−67.4	36.1	53.9
		270	1594.8	−292.4	36.1	233.9
CVRPTW	0.05	30	1572.8	9.88	227.6	−197.6
		90	1569.8	6.88	227.6	−137.6
		270	1565.0	−2.12	227.6	42.4
	0.25	30	1663.7	46.7	216.8	−186.8
		90	1648.7	31.7	216.8	−126.8
		270	1603.7	−13.3	216.8	53.2
	1.25	30	1756.0	209.0	197.2	−167.2
		90	1681.0	134.0	197.2	−107.2
		270	1456.0	−91	197.2	72.8

**Table 9:** The proportion of the cost when carbon price is 0.05.

CEVRPTW			CVRPTW		
Sub-Cost	Amount of Money	Proportion (%)	Sub-Cost	Amount of Money	Proportion (%)
F	1865.6	100	F	1574.3	100.0
$C_{1}$	300	16.08	$C_{1}$	300	19.1
$C_{2}$	886.2	47.50	$C_{2}$	190.7	12.1
$C_{3}$	612.9	32.85	$C_{3}$	444.7	28.2
$C_{4}$	64.5	3.46	$C_{4}$	627.5	39.8
$C_{5}$	1.86	0.10	$C_{5}$	11.4	0.72

**Table 10:** The proportion of the cost when carbon price is 0.25.

CEVRPTW			CVRPTW		
Sub-Cost	Amount of Money	Proportion (%)	Sub-Cost	Amount of Money	Proportion (%)
F	1874.3	100	F	1671.2	100
$C_{1}$	300	16.01	$C_{1}$	300	17.95
$C_{2}$	653.10	34.85	$C_{2}$	190.86	11.42
$C_{3}$	873.46	46.60	$C_{3}$	528.38	31.62
$C_{4}$	48.19	2.58	$C_{4}$	597.74	35.77
$C_{5}$	9.55	0.51	$C_{5}$	54.22	3.24

**Table 11:** The proportion of the cost when carbon price is 1.25.

CEVRPTW			CVRPTW		
Sub-Cost	Amount of Money	Proportion (%)	Sub-Cost	Amount of Money	Proportion (%)
F	1932.3	100	F	1793.5	100
$C_{1}$	300	15.53	$C_{1}$	300	16.73
$C_{2}$	628.10	32.51	$C_{2}$	165.24	9.21
$C_{3}$	913.95	47.30	$C_{3}$	538.13	30.00
$C_{4}$	45.13	2.34	$C_{4}$	543.60	30.31
$C_{5}$	45.125	2.34	$C_{5}$	246.52	13.75
